# Ginsenoside compound K-based multifunctional liposomes for the treatment of rheumatoid arthritis

**DOI:** 10.1080/10717544.2025.2464190

**Published:** 2025-02-16

**Authors:** Meng Zhang, Ru Zhang, Chunbo Feng, Xinnan Jiang, Xinchun Xu, Jianxin Wang

**Affiliations:** a Science and Technology Innovation Center, Guangzhou University of Chinese Medicine, Guangzhou, China; b Department of Pharmaceutics, School of Pharmacy, Fudan University & Key Laboratory of Smart Drug Delivery, Ministry of Education, Shanghai, China; c R&D Center, Shanghai Jahwa United Co., Ltd., Shanghai, China; d Shanghai Xuhui Central Hospital, Xuhui Hospital Attached to Fudan University, Shanghai, China

**Keywords:** Ginsenoside compound K, liposomes, rheumatoid arthritis, inflamed joints targeting, dexamethasone, synergistic effect

## Abstract

The clinical treatment of rheumatoid arthritis (RA) with first-line therapeutic drugs is hindered by the poor solubility, low bioavailability, off-target toxicity, and insufficient accumulation in inflamed joints. Liposomes have been shown to mitigate some of these limitations in drug delivery systems. However, the use of cholesterol to stabilize liposomal structures remains controversial due to its potential association with cardiovascular diseases. Here, we developed a novel liposome based on ginsenoside compound K (CK), which not only serves as an effective therapeutic agent for RA but also replaces cholesterol as a membrane stabilizer to address these challenges. Compared with conventional liposomes, ginsenoside CK Liposomes (CK@Lipo) are excellent nanoparticles, with CK stabilizing the liposomal structure and providing targeting functionality toward inflamed joints. When encapsulated with dexamethasone (Dex), CK@Lipo exhibits a synergistic anti-inflammatory effect, slowing the progression of RA. This study provides a theoretical basis for the future development of multifunctional novel ginsenoside CK@Lipo.

## Introduction

Rheumatoid arthritis (RA) is one of the most common immune-mediated diseases, characterized by progressive joint damage, typically involving the small joints of the hands and feet. It has a global prevalence of 0.5–1.0% and can lead to permanent disability in severe cases (Gravallese & Firestein, 2023). RA is mainly caused by a combination of genetics and specific environmental factors, such as the HLA-DR locus and cigarette smoke (Klareskog et al., 2006). Generally, RA proves challenging to completely cure, necessitating lifelong treatment (Finckh et al., 2022). In recent years, frontline drugs for the clinical management of RA have included non-steroidal anti-inflammatory drug (NSAID), glucocorticoids (GCs), disease-modifying antirheumatic drugs (DMARDs), and biological agent. Commonly used medications within these categories include ibuprofen (an NSAID), dexamethasone (a GC), methotrexate (a DMARD), and etanercept (a biological agent) (Aletaha & Smolen, 2018; Radu & Bungau, 2021). Nevertheless, the therapeutic efficacy of these drugs is hampered by many factors such as poor solubility, low bioavailability, off-target toxicity, and insufficient accumulation in inflamed joints (Abolmaali et al., 2013; Shen & Du, 2023). As a result, refining drug formulations and augmenting bioavailability have emerged as novel challenges in the field of RA drug research.

Currently, the development of nanotechnology has brought new perspectives, particularly through extensive research on liposomes (Radu & Bungau, 2023). Liposomes are spherical vesicles primarily composed of phospholipids and cholesterol, and have been widely applied in drug delivery owing to their favorable biocompatibility, low toxicity, and the capability to carry both hydrophilic and lipophilic drugs simultaneously (Akbarzadeh et al., 2013; Rahman et al., 2016). The phospholipid structure, resembling biological membranes, imparts biocompatible characteristics to liposomes, while cholesterol contributes to structural stability. However, prolonged use of cholesterol may activate the immune system by enhancing its interaction with complement system protein C3 (Ishida et al., 2000; Inglut et al., 2020). Therefore, it is imperative to further explore cholesterol substitutes for stabilizing liposomes.

**Figure 1.**
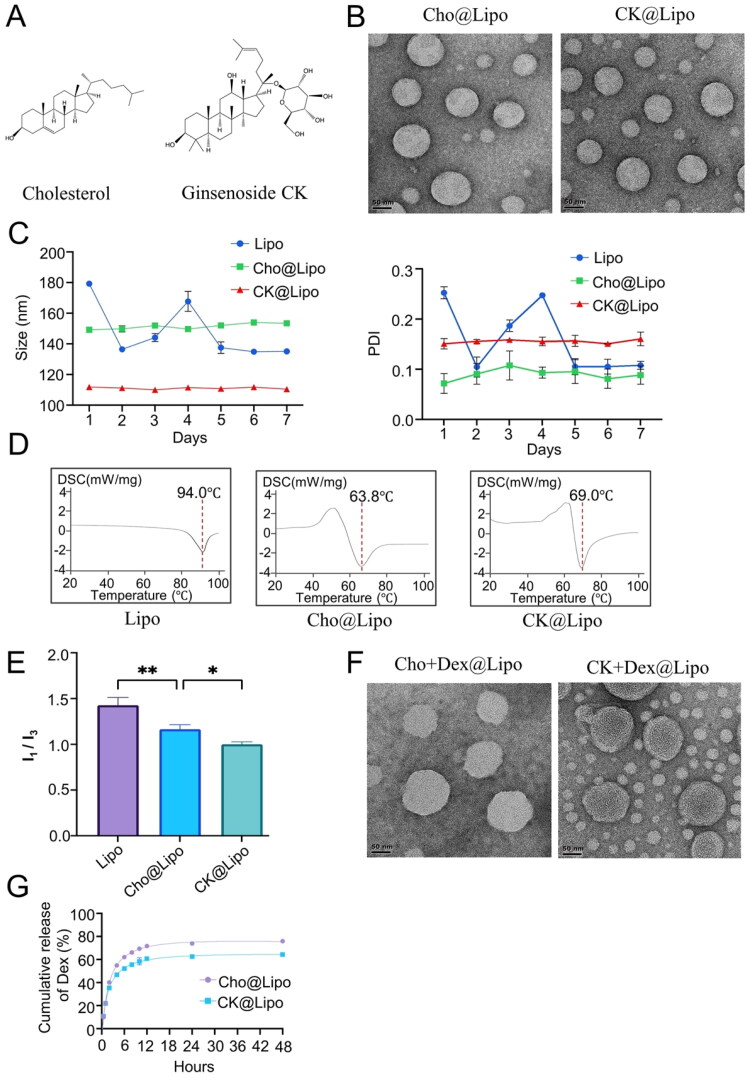
Characterization of liposomes. (A) Chemical structure of cholesterol and ginsenoside CK. (B) Transmission electron microscopy images of cholesterol liposomes (Cho@Lipo) and ginsenoside CK liposomes (CK@Lipo); scale bar = 50 nm. (C) Changes in size and polydispersity index of liposomes stored at 4 °C for seven days (*n* = 3; mean ± standard deviation). (D) Differential scanning calorimetric curves of Lipo, Cho@Lipo, and CK@Lipo. (E) Micro-polarity of liposome membranes. *p < .05, **p < .01. (F) Transmission electron microscopy images of dexamethasone-loaded cholesterol liposomes (Cho + Dex@Lipo) and dexamethasone-loaded ginsenoside CK liposomes (CK + Dex@Lipo); scale bar = 50 nm. (G) *In vitro* release of dexamethasone from liposomes.

The utilization of traditional herbs for treating chronic diseases has emerged as a new focal point, with ginseng being a notable example (Liu et al., 2022). Ginsenosides, the primary active components in ginseng, has undergone extensive research, uncovering various functions such as anti-inflammatory properties (Yi, 2019), attenuation of myocardial ischemia (Jiang et al., 2021), and immunomodulation (You et al., 2022). As a result, many ginsenosides have found broad applications in the pharmaceutical and functional food industries (Ratan et al., 2021). Ginsenoside compound K (CK), the main rare metabolite of many ginsenoside, not only has many pharmacological activities, such as anti-inflammation and anti-cancer, but also shares a chemical structure similar to cholesterol, characterized by a sterol structure (Yang et al., 2015; Hong et al., 2019) (Figure 1(A)). Previous studies have also demonstrated the ability of ginsenosides to modify membrane fluidity (Francis et al., 2002; Tsuchiya, 2015). Therefore, it was boldly hypothesized that ginsenoside CK may have the potential to replace cholesterol and play a role in stabilizing liposomes. Moreover, prior researches indicate a significant intervention effect of ginsenoside CK on RA (Zhang et al., 2021). As Chen et al. found that CK alleviates arthritis in rats by inhibiting T cell activation (Chen et al., 2014). Wang et al. discovered that CK improves arthritis by suppressing glycolysis in synovial cells through the NF-κB/HIF-1α pathway (Wang et al., 2023). Although the anti-RA effects of ginsenoside CK have been proved *in vitro* and *in vivo*, its development has been hindered by poor solubility and limited accumulation at RA sites following intravenous administration. Therefore, it is of great significance and importance to develop new delivery system of CK to realize its RA targeting distribution and exert better therapeutic efficacy. Previous studies have shown that the vascular permeability of inflamed tissues increases during the RA. Based on this, liposomes can passively target and remain in joint sites, thus enhancing the therapeutic efficacy of drugs (van den Hoven et al., 2011).

In the present study, a novel ginsenoside CK liposome (CK@Lipo) was prepared by taking it as an anti-RA drug and a membrane modulator simultaneously. To enhance the pharmacological effect, dexamethasone (Dex), a commonly used anti-RA drug, was encapsulated to achieve synergistic effect with CK. The liposomes were systematically compared with conventional cholesterol liposomes both *in vivo* and *in vitro* in the models of RA.

## Materials and methods

### Materials

Egg yolk lecithin (PL-100M) was purchased from Shanghai AVT Pharmaceutical Technology Co. (Shanghai, China). Ginsenoside CK was a gift from Zhejiang Hisun Pharmaceutical Co. (Taizhou, China). Cholesterol, anhydrous ethanol, dimethyl sulfoxide (DMSO), dichloromethane, and 1,6-diphenyl-1,3,5-hexatriene (DPH) were obtained from Sinopharm Chemical Reagent Co. (Shanghai, China). Dexamethasone was purchased from Dalian Meilun Biotechnology Co., Ltd. (Dalian, China). 3-(4,5-Dimethyl-2-thiazolyl)-2,5-diphenyl-2H-tetrazolium bromide (MTT) was obtained from Sigma-Aldrich Company (St. Louis, MO).

### Preparation of liposomes

Liposomes were prepared using the thin film hydration method (Large et al., 2021). In brief, CK and EPC were added in a 3:10 (w/w) ratio, dissolved in a solvent mixture (dichloromethane:ethanol = 1:1), and thoroughly mixed. The mixture underwent evaporation under a vacuum using a rotary evaporator, forming a thin lipid film in a round flask. The thin film was hydrated with PBS at 48 °C for 30 minutes and then extruded through polycarbonate membranes with pore sizes of 0.8 µm, 0.4 µm, and 0.2 µm, respectively. Ginsenoside CK@Lipo was prepared and labeled as CK@Lipo. Cholesterol liposomes were prepared following the same procedure, but with an equivalent amount of ginsenoside CK replaced by cholesterol. Cholesterol liposomes were labeled as Cho@Lipo. Dex-loaded liposomes were prepared using the same method, with the addition of Dex during mixing process with a mass ratio of 1:20. Dex-loaded ginsenoside CK@Lipo was labeled as CK + Dex@Lipo, while Dex-loaded cholesterol liposomes were labeled as Cho + Dex@Lipo.

### Characterization of liposomes

The particle size, polydispersity index (PDI) and zeta potential of liposomes were determined by dynamic light scattering technique of Zetasizer Nano ZS90 (Nano ZS90, Malvern Instruments, Malvern, UK). Each sample was measured in triplicate.

The stability of the vehicles was assessed by measuring the changes in particle size and PDI of liposomes stored at 4 °C for seven consecutive days.

The morphology of the liposomes was visualized using transmission electron microscopy (TEM, Tecnai G^2^ F20 S-TWIN, FEI, Hillsboro, OR). Prior to imaging, the liposomes adsorbed on the copper mesh were stained in the dark with 2% uranyl acetate for 30 minutes.

### Differential scanning calorimetry (DSC)

Thermal analysis was performed to observe the heat effect difference of liposomes with a differential scanning calorimeter (DSC8000, PerkinElmer, Waltham, MA) (Biltonen & Lichtenberg, 1993). Aliquots of samples were placed in aluminum trays and sealed by a tablet press. Heating scans were performed from 20 to 100 °C at a rate of 10 °C/min under a dry nitrogen fill and a baseline was recorded using a blank sealed aluminum disk.

### Micro-polarity of liposome membranes

A fluorescence spectrophotometer (FS5, Techcomp, Kwai Chung, Hong Kong, China) and pyrene probe were used to measure the micro-polarity of membranes. A solution of 2 mM pyrene–acetone was mixed with liposomes at a ratio of 1:500 and incubated at 4 °C for 12 hours. The excitation slit (Ex) and emission slit (Em) were both set to 2.5 nm, while the excitation voltage was maintained at 700 V with an excitation wavelength of 338 nm. After excitation, pyrene displayed five absorption peaks. The fluorescence intensities of the first peak at 373 nm (*I*_1_) and the third peak at 384 nm (*I*_3_) were measured. The micro-polarity of the phospholipid bilayer surrounding pyrene is closely related to the fluorescence intensity ratio (*I*_1_/*I*_3_), with smaller ratios indicating lower micro-polarity in the pyrene probe environment (Lee et al., 2019; Tai et al., 2019).

### Determination of encapsulation efficiency

The encapsulation efficiency (EE%) of Dex or CK in liposomes was assessed using the ultrafiltration method. Liposomes were placed in ultrafiltration tubes with a 20 kDa molecular weight cutoff (Millipore, Burlington, MA) and centrifuged at 10,000 × *g* for five minutes at 4 °C. The lower layer was collected, and the content of unencapsulated Dex or CK was determined using high-performance liquid chromatography (Agilent Technologies, Santa Clara, CA). For Dex determination, the mobile phase comprised 30% deionized water and 70% methanol, with a flow rate of 1 mL/min, column temperature set at 25 °C, and detection wavelength at 240 nm, following previous protocols (Jia et al., 2018). CK determination employed a mobile phase of acetonitrile:water = 55:45, with a flow rate of 1 mL/min, column temperature set at 35 °C, and detection wavelength at 203 nm. The EE% was calculated by the following formula:

$$\text{EE}\%=\frac{\text{the total weight of drug}-\text{weight of unencapsulated drug}}{\text{the total weight of drug}}\times 100\%$$

### *In vitro* release of Dex from liposomes

Drug release was characterized *in vitro* using a dialysis approach (Nkanga et al., 2019). In brief, 1 mL of Cho + Dex@Lipo and CK + Dex@Lipo were loaded into dialysis bags (Mv = 20 kDa) in 50 mL of release medium with PBS solution containing 0.5% SDS and shaken at 37 °C at 100 rpm. Samples were obtained at 0, 0.5, 1, 2, 4, 8, 12, 24, and 48 h, respectively, while the dialysis strip was transferred to fresh medium. The amount of Dex in the medium was detected by HPLC and the curve of liposomal drug release over time was plotted.

### Cell lines

HUVEC cells (human umbilical vein endothelial cells) were sourced from the Cell Bank of the Chinese Academy of Sciences (Shanghai, China), with CVCL number CVCL_2959 and catalog number CRL-1730. The cells were cultured in DMEM medium supplemented with 10% fetal bovine serum and 1% penicillin–streptomycin, and incubated at 37 °C in a 5% CO_2_ environment.

### Cell proliferation inhibition

MTT assay was performed to detect the inhibitory effect of various drugs on inflammation-induced HUVEC cells. In brief, HUVEC cells induced by 10 ng/mL TNF-a were seeded into a 96-well plate at a density of 5000 cells per well. After adherence, the cells were exposed to 100 µL of each drug solution for 24 hours. The drug concentrations were prepared starting from 125 µg/mL and serially diluted twofold to a minimum concentration of 0.98 µg/mL. After the treatment period, MTT solution was added to each well and incubated for four hours, with the final working concentration of MTT being 0.5 mg/mL. After four hours, the culture medium was discarded, and the formazan crystals were fully dissolved in DMSO. The optical density (OD) was then measured at 570 nm using a microplate reader. Cell viability (%) was calculated by the formula:

$$\text{Cell viability }(\%)=\frac{\text{OD }(\text{experimental group})-\text{OD }(\text{blank})}{\text{OD }(\text{control group})-\text{OD }(\text{blank})}\times 100\%$$

### Cell migration assay

HUVEC cells were seeded in six-well plates at a density of 5 × 10^5^ cells per well and stimulated with 10 ng/mL TNF-a after the cells were adherent. When cell confluence reached approximately 90%, a wound was created using a pipette tip, and the cells were co-incubated with different drugs. The final concentrations of CK and Cho were 18 µg/mL, while Dex was used at 3 µg/mL. Cell migration to the wound surface was observed and photographed at 6 h and 12 h. The migration area was calculated using Image J software (Bethesda, MD).

### Cellular uptake *in vitro*

HUVEC cells were seeded at a density of 10,000 cells/well in a 12-well plate. After adhering, the cells were induced with 10 ng/mL TNF-a for 12 hours. Following induction, DiD-labeled Chol@Lipo and CK@Lipo were added separately to the cells for co-incubation, with a working concentration of 20 μg/mL for DiD. After four hours, the cells were stained with DAPI for 10 minutes and washed three times with PBS. Subsequently, the samples were visualized and captured using confocal microscopy (Carl Zeiss, Oberkochen, Germany), and quantitative analysis with flow cytometry (FACS Calibur, BD Biosciences, Franklin Lakes, NJ).

Additionally, to investigate uptake mechanisms, after TNF-a stimulation, GC receptor antagonists RU486 at a concentration of 0.5 µM and GC receptor small interfering RNA (GRsiRNA) at a concentration of 40 pmol were separately added to HUVEC cells for 12 hours of co-incubation, followed by the same procedures as mentioned above.

### Animal

The experiment was performed after approval from the Ethics Committee for Laboratory Animal Care of Fudan University (Ethics No. 2021-09-YJ-WJX-98). Male DBA/1 mice, aged 8 weeks and weighing 18 g ± 2 g, were purchased from Slaccas Laboratory Animal Co., Ltd. (Shanghai, China). All 46 mice were maintained at 24 °C and 65 ± 15% relative humidity with 12-hour light–dark cycle. After one week of adaptive feeding, five mice were randomly selected as normal mice, and the rest were used to induce the collagen-induced arthritis (CIA) model. A 20% loss in body weight or the inability of mice to feed themselves was established as the humane endpoint for this experiment (Hawkins et al., 2015). All animal experiments were conducted between October 8 2021 and December 17 2022.

### Induction and assessment of CIA

The CIA mouse model is one of the most widely utilized animal models for investigating RA. This model shares several pathological features with RA, including synovial hyperplasia, mononuclear cell infiltration, and cartilage degradation (Brand et al., 2007). Typically, autoimmune arthritis in DBA/1 mice is induced using an emulsion of type II collagen (CII) and complete Freund’s adjuvant. Based on these characteristics, the CIA-induced DBA/1 mouse was selected as the model for this study.

The procedure for the induction and assessment of CIA is outlined as follows: bovine CII was dissolved in 0.1 mol/mL acetic acid before being fully emulsified with an equal volume of complete Freund’s adjuvant in an ice water bath. Isoflurane anesthesia was administered during the procedure, with an induction dose of 4% and a maintenance dose of 1.5%. Under anesthesia, DBA/1 mice were subcutaneously injected with 100 µL of the above emulsion at the base of the tail, and the procedure was repeated 21 days later (Brand et al., 2007). From day 28, the arthritis index of the mice was assessed and quantified on a scale of 0–4. The scoring criteria were as follows: score 0: no inflammation; score 1: one toe red and swollen; score 2: two or more toes swollen but paws not swollen; score 3: three toes swollen or extending into the paw moderately swollen and red; score 4: severe swelling of the entire paw accompanied by the ankle joint (Brand et al., 2007).

### *In vivo* imaging in RA mice

The targeting ability of liposomes to inflamed joints and biodistribution were assessed *in vivo* in CIA-induced DBA/1 mice and visualized by near-infrared fluorescence (NIRF). Briefly, six mice were divided into two groups of three mice each according to their arthritis index and then randomly assigned to treatment groups. 200 µL of DiR-labeled liposomes were injected into the tail vein of the mice. Images were captured at 1, 2, 4, 6, 12, and 24 hours post-injection, with the mice under isoflurane anesthesia during imaging. After 24 hours, the mice were euthanized by cervical dislocation, and major organs and hind limbs were collected for imaging.

### Treatment of RA mice

After the establishment of RA model, the mice with similar scores were randomly divided into seven groups, each containing five mice. They were administered equal volumes of PBS, free Dex, free Dex + CK, CK@Lipo, Cho@Lipo, CK + Dex@Lipo, and Cho + Dex@Lipo, respectively. The dosage of CK or Cho in the liposomes was 30 mg/kg, while Dex was administered at 5 mg/kg. Mice in each group received a tail vein injection every two days. The arthritis index was recorded by two independent observers who were blinded to the treatment regimens. After 15 administrations, the mice were euthanized, and their limbs and the blood were collected for histological examination and enzyme-linked immunosorbent assay (ELISA) analysis.

### Cytokine levels assay

The serum levels of TNF-α in the mice were measured using an ELISA kit (Mlbio, Shanghai, China, catalog number: ml002095). Serum was obtained by centrifuging the blood at 1000 × *g* for 10 min. After diluting the serum with a diluent, 50 µL was added to the reaction wells of the assay kit, followed by an equal volume of horseradish peroxidase-conjugated antibody. The mixture was incubated at 37 °C in the dark for one hour. After washing the reaction wells three times, 50 µL of substrate A and 50 µL of substrate B were added to each well and incubated at 37 °C in the dark for 15 minutes. Then, 50 µL of stop solution was added to each well, and the OD value was immediately measured at 450 nm using a microplate reader. The corresponding concentration of each sample was calculated based on the standard curve.

### Histological studies

The collected limbs were decalcified with PBS containing 15% EDTA for 1 month, embedded in paraffin and sectioned at a thickness of 4 μm. Sections were stained with hematoxylin–eosin (HE) for further observation of the structural changes in the joint tissues.

### Statistical analysis

The results were analyzed using GraphPad Prism 9.0 software (La Jolla, CA), and experimental data are represented as mean ± standard deviation (SD). Comparisons between two groups are conducted using *t*-tests, while comparisons among multiple groups are performed using one-way analysis of variance (ANOVA). A *p* value <.05 indicates statistical significance.

## Results and discussion

### Characterization of liposomes

Size, PDI, and zeta potential are three important indicators to evaluate the homogeneity and stability of liposomes (Guimarães et al., 2021). Size refers to the average diameter, which affects the circulation time and bioavailability of liposomes *in vivo* (Alexis et al., 2008). PDI reflects the dispersion of the vehicles, and values less than 0.3 indicate good homogeneity (Danaei et al., 2018). Zeta potential is considered to be the fundamental physical property that controls the electrostatic interactions between suspended particles (Kaszuba et al., 2010).

The size of liposome affects its circulation time and distribution in the organism, so the appropriate size of liposome can further improve the bioavailability. Hong et al. reported that liposomes with a particle size of 100 nm exhibited superior pharmacokinetic parameters compared to those with sizes of 70 nm, 200 nm, and 350 nm. This was attributed to the fact that larger particles were more readily recognized and phagocytosed by the reticuloendothelial system, while smaller liposomes were more easily absorbed by the liver and filtered by the kidney (Ren et al., 2019).

The results of the characterization of liposomes with different components are shown in Table 1. The average particle size of pure phospholipid liposomes was about 180 nm. Upon the addition of Cho or CK, the particle size and PDI of the liposomes significantly decreased, indicating that these stabilizers may compact the liposomal structure, thereby reducing particle size and improving uniform dispersion. The particle size of CK@Lipo was approximately 111 nm, close to the optimal size of 100 nm as reported, while Cho@Lipo exhibited a slightly larger size of around 150 nm. The zeta potentials of the two carriers were around −30 mV, which is higher than the −17 mV observed for pure phospholipid liposomes. Studies have shown that liposomes with strongly negative zeta potentials exhibit repulsive forces in the medium, preventing natural aggregation tendencies (Laouini et al., 2012). Therefore, it can be concluded that CK effectively stabilizes liposomes and ensures uniformity in their physicochemical properties.

**Table 1.** Characterization of blank liposomes and dexamethasone-loaded liposomes (*n* = 3; mean ± standard deviation).

	Size	PDI	Zeta potential	EE% of CK	EE% of Dex
Lipo	179.30 ± 0.46	0.25 ± 0.01	−17.81 ± 0.69	–	-
Cho@Lipo	149.23 ± 0.40	0.07 ± 0.02	−33.33 ± 1.02	–	-
CK@Lipo	111.87 ± 0.80	0.15 ± 0.01	−29.36 ± 1.65	90.32 ± 2.18	-
Cho + Dex@Lipo	157.07 ± 0.58	0.06 ± 0.01	−32.26 ± 0.46	–	90.77 ± 2.41
CK + Dex@Lipo	119.77 ± 1.17	0.07 ± 0.03	−31.33 ± 0.61	87.88 ± 2.05	87.84 ± 2.72

### Liposome morphology

The TEM images of the two types of liposomes are shown in Figure 1(B). The results indicated that Cho@Lipo appear spherical, with smooth surfaces and uniform dispersion. The particle size was consistent with the measurements obtained from the Zetasizer. Similarly, liposomes prepared with ginsenoside CK replacing cholesterol exhibited no significant difference in morphology compared to traditional liposomes. This confirms the feasibility of ginsenoside CK as a stabilizer for liposomal membranes.

### Stability

The uniformity and stability of the liposomes were evaluated by measuring the changes in their size of liposomes over time. The results of liposomes stored continuously at 4 °C for seven days are shown in Figure 1(C).

The particle size and PDI of Lipo prepared with EPC alone showed a wide range of fluctuations over time, while those of CK@Lipo and Cho@Lipo remained relatively stable. It is speculated that liposomes without stabilizers may undergo phenomena such as fusion and swelling, leading to an increase in size and drug leakage. The storage stability of liposomes is crucial for clinical application, as formulations with uniform particle size and distribution can exert good and stable therapeutic effects. Compared with Cho@Lipo, CK@Lipo demonstrated smaller variations in particle size and PDI over seven days, providing ample evidence for the feasibility of ginsenoside CK as a liposome stabilizer.

### DSC analysis

The phase transition temperature (*T*_m_) of phospholipids refers to the temperature at which phospholipids transition from the gel to the liquid crystal phase, which mainly depends on factors such as the length of the fatty acid chain and the nature of the polar external groups (Hussain et al., 2017; Zamani et al., 2018), and thus affects the fluidity of the lipid bilayer. Below *T*_m_, phospholipids are in the gel phase, exhibiting low mobility and low permeability. In contrast, near *T*_m_, the permeability of the lipid bilayer significantly increases due to the presence of a highly permeable interfacial region between the coexisting gel and liquid crystal phase domains (Beltrán-Gracia et al., 2019).

Figure 1(D) displays the DSC thermograms of Lipo, Cho@Lipo, and CK@Lipo in the temperature range of 20–100 °C. The main heat absorption peak of Lipo was observed at 94 °C, corresponding to the phase transition temperature of phospholipids. After the addition of Cho and CK, the phase transition temperature decreased to 63.8 °C and 69 °C, respectively. It is hypothesized that ginsenosides and cholesterol share the same steroidal ring parent nucleus, which interacts with the hydrocarbon chains of phospholipids during liposome preparation, altering the thermal properties of the phospholipid bilayer and thereby improving the permeability and mobility of the liposomes at lower temperatures.

### Micro-polarity of liposome membranes

Pyrene, a sensitive probe for micro-polarity, is commonly employed to assess the micro-polarity of liposomal membranes. Its fluorescence emission spectrum displays five primary absorption peaks spanning 370–430 nm. In comparison with the third absorption peak at 383 nm, the intensity of the first absorption peak at 373 nm increases with the escalating polarity of the solvent. Consequently, the intensity ratio (*I*_1_/*I*_3_) is typically utilized to represent polarity changes within the microenvironment. A smaller ratio signifies a lower micro-polarity within the microenvironment (Mazor et al., 2012). Simultaneously, the polarity represented by *I*_1_/*I*_3_ can also reflect the intermolecular forces within the liposomal membrane. A smaller ratio suggests a tighter association between the membrane material and phospholipid molecules, which can hinder water molecules from permeating the bilayer, thus render the liposomal structure more stable.

As shown in Figure 1(E), among the three groups, the Lipo group exhibited the highest *I*_1_/*I*_3_ value, indicating that the polarity of pure phospholipids was the largest. With the addition of Cho or ginsenoside CK, the fluorescence ratio decreased significantly, proving that the incorporation of membrane materials reduced the micro-polarity of liposomes. This finding suggests a potential interaction between the membrane materials and phospholipid molecules, leading to a possible reduction in the proportion of hydrophilic domains. Moreover, compared to the Cho@Lipo group, the CK@Lipo group showed a slightly smaller fluorescence ratio, indicating that ginsenoside CK had a superior binding affinity and tighter association with phospholipid molecules than cholesterol, thereby providing a more stable encapsulation area for hydrophobic molecules.

### Characterization of Dex-loaded liposomes

Dex is a type of GC medication commonly used to treat RA in clinical settings. However, due to the serious side effects associated with long-term or high-dose usage of free drugs, the application of Dex is greatly restricted (Black & Grodzinsky, 2019). Therefore, it is necessary to develop new formulations of Dex to improve its efficacy and reduce side effects. Herein, Dex is used as a model drug to exert a synergistic effect with ginsenoside CK to treat RA.

The results in Table 1 show that the addition of Dex caused a minor variation in particle size compared to blank liposomes, with an increase of approximately 8 nm. The PDI and zeta potential values remained within a stable range. The EE% of Dex in CK@Lipo was about 88%, similar to that in Cho@Lipo, while the EE% of CK in CK@Lipo was approximately 88%.

TEM results in Figure 1(F) show that both CK@Lipo and Cho@Lipo maintained a spherical shape and uniform dispersion after the loading of Dex. Encapsulation efficiency and stability are crucial for drug protection and *in vivo* delivery. High EE% helps minimize wastage of expensive drugs and improves bioavailability, while stability is fundamental for keeping the efficacy and safety of new formulations. In summary, the results suggest that CK@Lipo demonstrates potential as a universal carrier based on its high encapsulation rate and good stability performance.

### *In vitro* release of Dex from liposomes

To prevent substantial drug loss prior to reaching the intended site of action, thereby averting potential adverse reactions, controlling the drug release rate from the formulation has emerged as a critical criterion for evaluating the carrier (Modi & Anderson, 2013). The *in vitro* release results illustrated in Figure 1(G) show that, under the same release conditions, both liposome groups exhibit an initial rapid release followed by a slower release phase. Two hours after the onset of release, differences in the release rates between the two groups became evident. The overall release amount of Dex from the CK + Dex@Lipo group was lower than that from the Cho + Dex@Lipo group. For instance, at 12 hours, the release rate of Dex from CK + Dex@Lipo was approximately 60.73%, whereas it reached 71.74% in Cho + Dex@Lipo. At 48 hours, the released Dex from Cho + Dex@Lipo was approximately 1.2 times that from CK + Dex@Lipo. These results further demonstrate that the liposomes prepared with CK, rather than Cho, possess superior sustained-release properties, which may potentially reduce the side effects and enhance the targeted distribution of Dex.

### Cell proliferation inhibition

**Figure 2.**
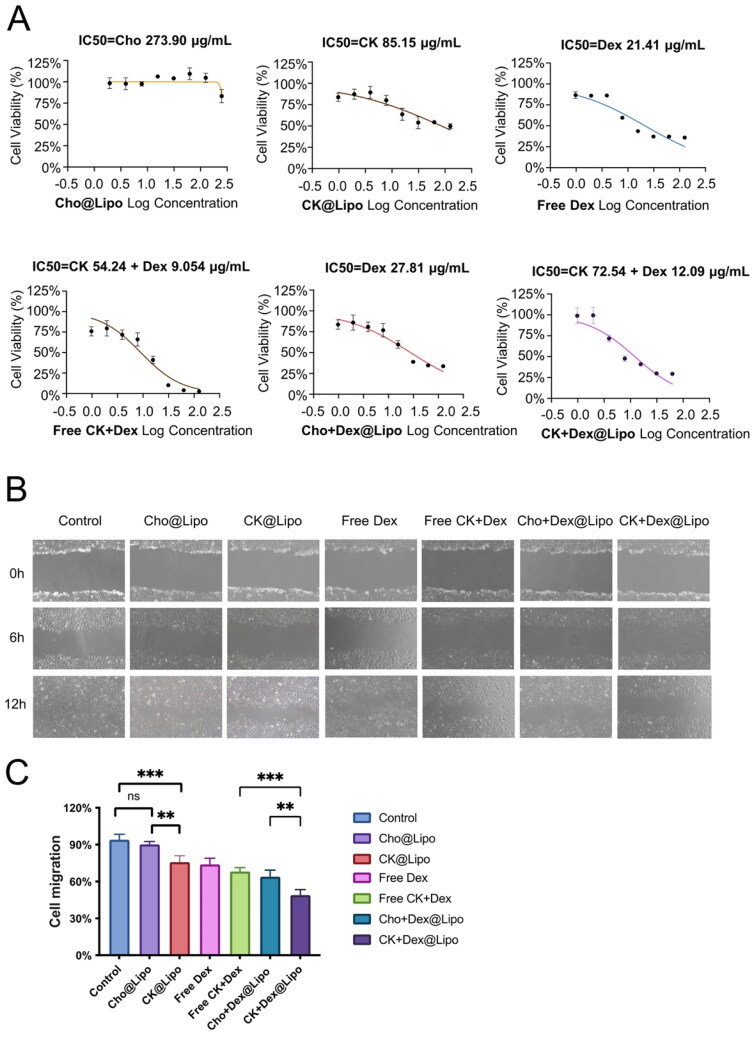
Results of cell proliferation inhibition and cell migration suppression by each treatment group. (A) The inhibitory effects of both liposomes and free drugs on inflamed HUVEC cells (*n* = 6; mean ± SD). (B) Representative image of HUVEC cells (×200). (C) The HUVEC cell migration rate in each group was calculated using Image J (Bethesda, MD) (*n* = 3; mean ± SD). **p < .01; ***p < .005; ns: no significance.

The data presented in Figure 2(A) reveal the inhibitory effects of both liposomes and free drugs on inflamed HUVEC cells. CK@Lipo achieved 50% inhibition of cell proliferation at a concentration of 85.15 μg/mL, whereas the Cho@Lipo group required a concentration of 273.90 μg/mL to achieve a similar effect, indicating that CK exhibits a significantly stronger inhibitory effect on cell proliferation compared to Cho. Free Dex induced approximately 50% cell death at a concentration of 21.41 μg/mL. However, when combined with free CK, the required concentration of Dex decreased to 9.054 μg/mL, demonstrating a synergistic effect between the two drugs and confirming that CK enhances the inhibitory effect of Dex on cell proliferation. Moreover, encapsulation of Dex in liposomes increased its IC_50_ value, suggesting that liposomal carriers provide a protective effect and mitigate the toxicity of Dex.

### Cell migration assay

In the RA environment, endothelial cells proliferate and migrate under the induction of inflammatory factors, leading to neovascularization in the synovial tissue (Feldmann & Maini, 2001). Therefore, inhibiting endothelial cell migration can effectively impede the malignant progression of RA. *In vitro* cell migration experiments were performed to simulate this pathological process (Giraudo et al., 1998). The results shown in Figure 2(B,C) demonstrate that under TNF-α stimulation, the migration ability of HUVEC cells is significantly enhanced. After 12 hours, the migration rate of the control group reached approximately 94%. However, co-incubation with Cho@Lipo showed no significant difference in the migration rate of HUVEC cells compared to the control group, suggesting that Cho@Lipo alone cannot inhibit HUVEC cells migration. On the contrary, CK@Lipo slowed down the migration rate of HUVEC cells, possibly due to the anti-inflammatory effects of CK. In addition, drugs containing Dex also exerted inhibitory effects on cell migration. Among these, the CK + Dex@Lipo group demonstrated the strongest impact on cell migration rate with a statistically significant difference compared to the Cho + Dex@Lipo group, indicating that the combined application of CK and Dex enhances anti-inflammatory effects. Furthermore, the migration rate of the CK + Dex@Lipo group was significantly lower than that of free CK + Dex, supporting the notion that the liposomal carrier enhances the bioavailability and efficacy of the medications.

### Mechanisms of cellular uptake and mechanism *in vitro*

The continuous proliferation of new blood vessels, known as ‘angiogenesis,’ in inflamed joints is considered a core factor in sustaining and promoting the development of RA. Disrupting angiogenesis not only deprives the inflamed area of necessary nutrients but also holds the potential to regress blood vessels and reverse the disease. Therefore, targeting blood vessels in RA has been considered an effective treatment strategy (Paleolog, 2002).

**Figure 3.**
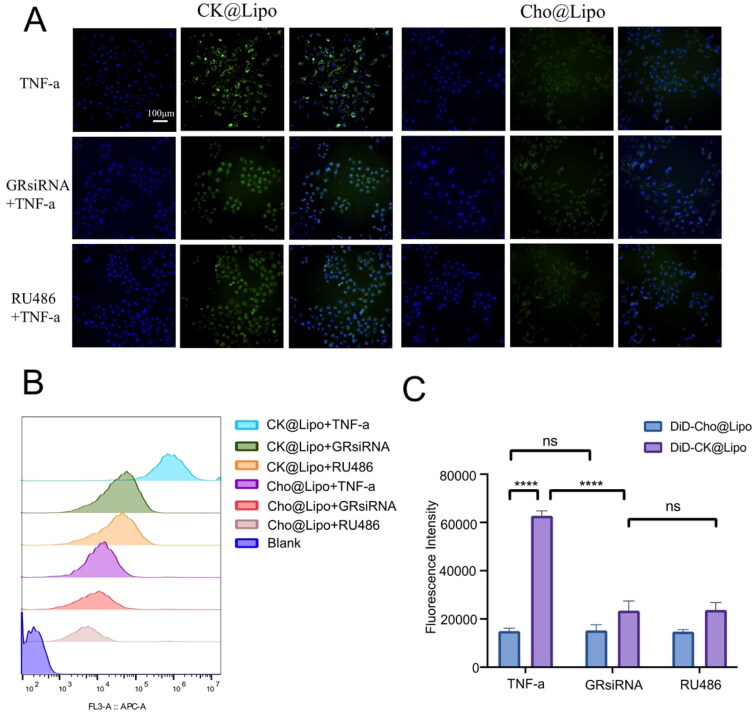
Cellular uptake of liposomes in HUVEC cells. (A) Confocal images of cellular uptake of liposomes in HUVEC cells (scale bar = 100 μm). (B) Analysis of cellular uptake mechanisms by flow cytometry. (C) Statistical analysis of cellular uptake results (*n* = 3; mean ± SD). ****p < .001; ns: no significance.

To evaluate the targeting efficacy of liposomes, the fluorescence intensity following the uptake of liposomes by TNF-α-induced HUVEC cells was measured. The results are depicted in Figure 3(A). Both DiD-labeled Cho@Lipo and CK@Lipo were internalized by inflamed cells. However, co-incubation with CK@Lipo resulted in significantly stronger fluorescence signals in HUVEC cells, with fluorescence intensity approximately 3.3 times higher than that observed with Cho@Lipo. This suggests that ginsenoside CK, as a substitute for cholesterol, significantly enhances the cellular uptake capability of liposomes.

Previous research has shown that the active component of ginsenosides, Rg1, can serve as a functional ligand for GC receptors and regulate the transcription of target genes when complexed with specific DNA sequences known as GC response elements (Lee et al., 1997; Du et al., 2011). Rg1 and Rg3 can also target HUVEC cells and modulate the expression of vascular endothelial growth factor (Mohanan et al., 2018). GC receptors belong to the nuclear receptor superfamily, which can respond to both natural corticosteroids and synthetic anti-inflammatory ligands, suppressing the inflammatory gene expression program. Therefore, they have become the main targets of anti-inflammatory treatment strategies (Valledor & Ricote, 2004; Flammer & Rogatsky, 2011). Based on these findings, we boldly hypothesize that the increase in cellular uptake may be mediated by GC receptors. To further confirm the hypothesis, inflamed HUVEC cells were treated with the GC receptor antagonists RU486 or GRsiRNA (Cadepond et al., 1997), and then co-incubated with DiD-labeled liposomes. Changes in cellular fluorescence intensity were observed and illustrated in Figure 3(B,C). The results showed that both RU486 and GRsiRNA treatments resulted in 60–70% decrease in cellular fluorescence intensity in the CK@Lipo group, while no significant changes were observed in the Cho@Lipo group. This indicates that cellular uptake in the CK@Lipo group is significantly influenced by GC receptor activity and can be facilitated by the highly expressed GC receptors on HUVEC cells. These results suggest that the substitution of Cho with ginsenoside CK in liposomes promotes cellular uptake by mediating interactions with GC receptors.

### Targeting and biodistribution of liposomes in RA mice

**Figure 4.**
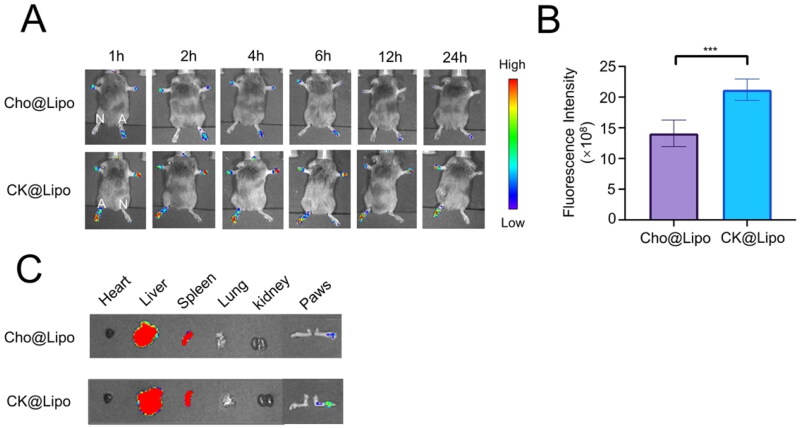
*In vivo* targeting and biodistribution of liposomes. (A) *In vivo* fluorescence imaging of normal (N) and inflamed paws (A, score >3) of CIA model mice after intravenous injection of different liposomes. (B) Relative fluorescence intensity of joints in each group (*n* = 3; mean ± SD). ***p < .005. (C) Fluorescence image of biodistribution of liposomes in CIA model mice at 48 h after injection.

The specific biodistribution of liposomes in mice illustrated in Figure 4 indicates that the liposomes labeled with DiR do not aggregate in the mice paws without inflammation but produce obvious fluorescence signals in arthritis joints. It is speculated that under RA pathological conditions, bone destruction accompanied by vascular opacities creates a high permeability akin to tumors, which facilitates the entry of nano-carriers into swollen joints (van den Hoven et al., 2011). Surprisingly, the fluorescence signals in the joints of mice treated with CK@Lipo were consistently stronger at each detection time point compared to those in the Cho@Lipo group, approximately 1.5 times greater, indicating a higher propensity for CK@Lipo to accumulate in inflamed joints. This finding corroborates with the trend observed in *in vitro* uptake experiments and confirms that replacing cholesterol with CK significantly enhances the targeted delivery of liposomes, likely due to their interaction with GC receptors.

### Therapeutic effect of liposomes on RA

**Figure 5.**
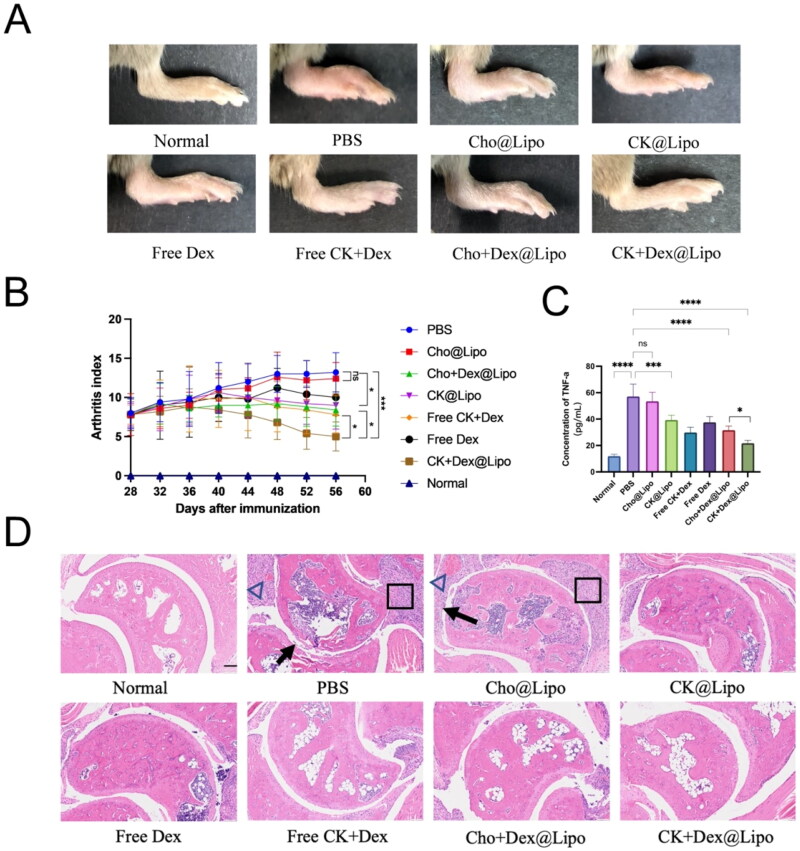
Therapeutic effects on RA mice. (A) Representative images of arthritic paws in different groups. (B) Arthritis index of mice in different groups after treatment (*n* = 5; mean ± SD). *p < 0.05; ***p < 0.005; ns, no significance. (C) The TNF-α levels in the serum of each group of mice (*n* = 5; mean ± SD). *p < 0.05; ***p < 0.005; ****p < 0.001; ns: no significance. (D) Pathological sections of hematoxylin and eosin stained ankle joints in each group (scale bar = 100 μm). The black arrows (→) indicate cartilage erosion, triangles (△) represent neovascularization, and squares (☐) denote synovial hyperplasia.

The effects of various pharmaceutical interventions on mice joints are depicted in Figure 5(A,B). Compared with the healthy mice in normal group, obvious swelling was observed in the joints of the mice in PBS group. After treatment with Cho@Lipo, no significant difference in arthritis indexes between the PBS group and Cho@Lipo group was found, indicating that Cho@Lipo alone is ineffective in treating RA. In contrast, CK@Lipo alleviated joint swelling and reduced arthritis indexes effectively, further validating that CK not only forms stable liposomes as a cholesterol substitute but also inhibits RA progression as a therapeutic agent. Furthermore, free Dex and all Dex-containing liposomes mitigated joint swelling to some extent and diminished arthritis indices, demonstrating the anti-RA effects of Dex. The joint index in the Cho + Dex@Lipo group was lower than that in the free Dex group, supporting the idea that liposomal encapsulation enhances the targeting efficiency and bioavailability of Dex, thereby strengthening its efficacy in RA intervention. Moreover, the combination of Dex and CK improved the anti-RA effect. Among all treatment cohorts, the mice in the CK + Dex@Lipo group displayed the lowest joint index, with the morphology of the metatarsal joints returning close to the normal. The anti-RA effect of CK + Dex@Lipo was significantly stronger than that of both the combination of free Dex with free CK and the Cho + Dex@Lipo, demonstrating that the co-administration of CK and Dex, along with the preparation of liposomes, is crucial for the treatment of RA.

### Cytokine levels

The TNF-α levels in mice serum were assessed after the treatment with different drugs, as illustrated in Figure 5(C). The results reveal that TNF-α levels in the serum of CIA mice treated PBS are significantly higher than those in healthy mice, indicating abnormal secretion of pro-inflammatory cytokines under diseased conditions. The Cho@Lipo carrier itself does not affect RA pathogenesis, as there is no significant difference in TNF-α levels between Cho@Lipo group and PBS group. Meanwhile, the substitution of cholesterol with CK led to a marked decrease in TNF-α levels, providing additional evidence of CK’s capability to suppress pro-inflammatory cytokine secretion. Both free and liposome-encapsulated Dex resulted in reduced TNF-α levels, and the combined application of CK and Dex exhibited a more pronounced effect than Dex alone. This finding further supports the notion of a synergistic enhancement between CK and Dex.

### Histopathological analysis of joints

As shown in Figure 5(D), the joint structure of normal mice is clear and intact, with evident joint space. In the PBS group and Cho@Lipo group, erosion of the joint surface cartilage, narrowing of the joint space, synovial hyperplasia infiltrated with inflammatory cells, and neovascularization were observed. Slight cartilage damage and synovial hyperplasia were found in the CK@Lipo and free Dex group, supporting their anti-inflammation effect. In the free CK + Dex group and Cho + Dex@Lipo group, joint synovial inflammation was obviously alleviated. Notably, the CK + Dex@Lipo group showed the least joint damage, characterized by evident joint space and cartilage morphology closest to that of normal mice.

## Conclusions

RA is a prevalent immune-mediated disease, primarily characterized by synovitis and bone erosion. Current RA treatments face significant challenges, including the need for high drug dosages, poor solubility, and inadequate targeting (Radu & Bungau, 2023). In light of these limitations, this study investigates the potential of liposomes as an advanced drug delivery system. Traditionally, liposomes incorporate cholesterol to stabilize the membrane structure. However, cholesterol presents certain risks, underscoring the importance of identifying safer alternatives in liposomal technology.

Ginsenoside CK has been identified as a promising candidate, not only for its therapeutic potential in RA but also due to its sterol structure, which closely resembles that of cholesterol. Consequently, ginsenoside CK has the potential to replace cholesterol, offering a dual function as both a drug delivery carrier and a therapeutic agent. To assess the feasibility of ginsenoside CK-based liposomes, Dex was selected as the encapsulated drug, and the synergistic effects of both compounds in RA treatment were evaluated. For the first time, a novel liposomal formulation of ginsenoside CK loaded with Dex was developed specifically for RA treatment. The results demonstrated that this formulation produced liposomes with uniform particle size, excellent stability, and high EE%, exhibiting a sustained release profile superior to that of conventional cholesterol-based liposomes. Ginsenoside CK not only stabilizes the liposomal membrane but also enhances membrane fluidity and micro-polarity. Moreover, through its interaction with GC receptors, ginsenoside CK facilitates increased cellular uptake and promotes the accumulation of liposomes in the inflamed joints of mice. *In vitro* experiments further indicated that ginsenoside CK-loaded Dex liposomes significantly inhibited the migration of HUVEC cells. In collagen-induced RA mouse models, this formulation markedly alleviated arthritic symptoms. These therapeutic effects likely arise from the synergistic anti-inflammatory actions of ginsenoside CK and Dex.

Building upon these findings, future research should focus on elucidating the molecular mechanisms underlying the interaction between ginsenoside CK and GC receptors, particularly how this interaction enhances cellular uptake and liposome aggregation within inflamed joints. Optimizing the ratio of Dex encapsulation and ginsenoside CK concentration will also be critical in determining the dose-dependent efficacy of the liposomal formulation for RA treatment. Furthermore, it is essential to explore the broader applicability of this formulation across different RA patient populations and address potential challenges related to its clinical translation.

This study provides robust evidence supporting the feasibility of replacing cholesterol with plant sterols like ginsenoside CK in the formulation of stable liposomes. Additionally, it offers new insights into the potential of combining natural plant products with steroid drugs in RA therapy. This innovative strategy paves the way for the future development of plant-based nanocarriers and combination therapy approaches.

## Data Availability

The data that support the findings of this study are available on request from the corresponding author. Ginsenoside compound K (CK) can replace cholesterol to form stable novel liposomes, targeting glucocorticoid receptors on endothelial cells of inflammation, thereby ameliorating symptoms of rheumatoid arthritis in mice. Ginsenoside CK achieves triple action as a drug, targeting moiety, and excipient.
